# Bruchid egg induced transcript dynamics in developing seeds of black gram (*Vigna mungo*)

**DOI:** 10.1371/journal.pone.0176337

**Published:** 2017-04-27

**Authors:** Indrani K. Baruah, Debashis Panda, Jagadale M.V, Deba Jit Das, Sumita Acharjee, Priyabrata Sen, Bidyut Kumar Sarmah

**Affiliations:** 1 DBT-AAU Centre, Assam Agricultural University, Jorhat, Assam, India; 2 Distributed Information Centre, Department of Agricultural Biotechnology, Assam Agricultural University, Jorhat, Assam, India; 3 Department of Agricultural Biotechnology, Assam Agricultural University, Jorhat, Assam, India; National Institute of Plant Genome Research, INDIA

## Abstract

Black gram (*Vigna mungo*) seeds are a rich source of digestible proteins, however, during storage these seeds are severely damaged by bruchids (*Callosobruchus spp*.), reducing seed quality and yield losses. Most of the cultivated genotypes of black gram are susceptible to bruchids, however, few tolerant genotypes have also been identified but the mechanism of tolerance is poorly understood. We employed Suppression Subtractive Hybridization (SSH) to identify specifically, but rarely expressed bruchid egg induced genes in black gram. In this study, Suppression Subtractive Hybridization (SSH) library was constructed to study the genes involved in defense response in black gram against bruchid infestation. An EST library of 277 clones was obtained for further analyses. Based on CAP3 assembly, 134 unigenes were computationally annotated using Blast2GOPRO software. In all, 20 defense related genes were subject to quantitative PCR analysis (qPCR) out of which 12 genes showed up-regulation in developing seeds of the pods oviposited by bruchids. Few major defense genes like defensin, pathogenesis related protein (PR), lipoxygenase (LOX) showed high expression levels in the oviposited population when compared with the non-oviposited plants. This is the first report on defense related gene transcript dynamics during the bruchid-black gram interaction using SSH library. This library would be useful to clone defense related gene(s) such as defensin as represented in our library for crop improvement.

## Introduction

Black gram [*Vigna mungo* (L) Hepper] is widely grown in South and South Asia, including India. The crop is grown for its highly proteinaceous seeds and is an important dietary component, especially in Indian Sub-continent which is dominated by the vegetarian population. Bruchid beetle, *Callosobruchus spp*. (Coleoptera: Bruchidae) causes considerable damage to both quantity and quality of black gram seeds during storage [1]. The primary infestation by bruchids begin in the field where insects lay eggs on pod wall and the larvae bore inside the mature pods and eat developing seeds. The adult beetles emerge at the time of plant maturity or soon afterwards. The secondary infestation occurs in the storage where adults emerging soon after harvest oviposit on the dry seeds and later, larvae bore inside the seeds to complete their life cycle [2]. Under conducive storage conditions, bruchid infestation causes total destruction of the seeds within six months [3]. The defense response in black gram due to bruchid infestation has not yet been elucidated, although there are reports on availability of both mild resistant genotypes and wild relatives with complete resistance to bruchids [4].

Eggs laid by insects on the plant’s tissue surface indicate impending damage by the emerging larvae [5]. Thus, plant induces both direct and indirect defense responses against eggs and prepares to protect itself from the emerging larvae. Interestingly, eggs encounter many aggressive responses by the plants that mainly target them and the emerging larvae rather than the female that lays eggs [6]. One of the interesting responses is that the plant is forming necrotic lesions surrounding the eggs that are laid to desiccate them. The hypersensitive response (HR) is a common response to any stress producing Reactive Oxygen Species (ROS) in the lesions [7,8,9]. The other responses observed in the plant after egg laying are, growth of neoplastic tissues to cast off eggs [10] and production of ovicidal substances [6] to kill the eggs as part of direct defense against insect.

Indirect defense also plays a critical role because of the release of volatile substance from the leaves that attract parasitoids to kill the eggs [11]. The defense response is triggered by the elicitors that are produced as exocrine secretions coating the eggs. The chemical composition of these secretions has also been characterized in a few instances. In the case of bruchids (*B*. *pisorum*, *C*. *maculate)*, bruchins which are 3-hydroxypropanoate esters of long-chain α, ω-diols are the potent elicitors. The elicitors play a major role in activating defensive response gene in the plants. Similarly, eggs also elevated the levels of defense responsive phytohormones, jasmonic acids (JA) and salicylic acid (SA). Both JA and SA are known to up-regulate defense transcripts in plants due to egg laying and feeding by larvae. These responses are also known as herbivore associated molecular patterns (HAMPs), which are specific plant indirect defense responses to specific herbivore-derived elicitors in oral/ovipositor secretions [12]. Thus, in most cases plant responds to egg deposition by herbivores [5].

This is the first report on changes in the transcript pattern in developing seeds of black gram induced after bruchids oviposit on its pods. As such, a functional genomics approach to study indirect resistance in black gram to confer resistance to stored grain pest, bruchid beetle (*Callosobruchus chinensis*), will allow the identification of genes expressed and validate their levels of expression during defense against attack. Analyses of the expressions and functions of pest inducible genes will facilitate understanding of the molecular mechanisms underlying the pest resistance by tolerant varieties. In the present study, a mild tolerant variety (IC8219) of black gram reported to resist bruchid infestation by 33.7–42% [4] was used to understand the defense mechanism against bruchids using Suppression Subtractive Hybridization (SSH). A SSH library was developed to identify differentially expressed genes in the developing seeds after oviposition by bruchids on the pod wall. The method is useful to identify tissue-specific gene expression and will help identify genes that are induced to resist bruchid infestation [13]. Considering SSH can be used to study plants for which limited sequence information is available, we have made an attempt to generate an SSH library of developing seed of pods oviposited by bruchids. Interestingly, we found changes in the transcript dynamics in the developing seeds and most of the transcripts identified were defense related.

## Materials and methods

### Plant material

The mild tolerant variety, IC8219, of Black gram was obtained from Indian Institute of Pulse Research (IIPR), Kanpur, India and was used for construction of the SSH cDNA library. Seeds were sown in the greenhouse of DBT-AAU Centre, AAU, Jorhat. Bruchids were obtained from Department of Entomology, AAU, Jorhat and were reared in laboratory conditions of 27°C, 70% R.H at DBT-AAU Centre, Jorhat. Both male and female adult bruchids are in controlled conditions in the greenhouse for 7–10 days. Bruchid eggs usually hatch after 3–5 days and emerging larvae bore inside the pod to eat the developing seeds, therefore, pods were collected after 7^th^ day of bruchid ovipositioning [14]. Immature pods that were oviposited by bruchids were collected after 7 days along with immature pods from the non-oviposited controls and stored at -80°C for further experimental use.

### DAB assay

Pods of black gram after 7 days of oviposition by bruchid beetles were excised and dipped overnight in 3,3’ Diamino benzidine (DAB) solution (1mg/ml; pH 3.6) at room temperature following the protocol described previously [15].

### Construction of SSH library and sequencing

The total RNA was extracted from the immature developing seeds of both bruchid egg laid and unlaid pods by PureLink^™^ Plant RNA reagent (Ambion,USA) as per manufacturer’s instructions. The total RNA from oviposited sample was considered as tester and total RNA from non-oviposited sample was considered as driver sample for SSH study. Double stranded cDNA was synthesized using SMARTER^™^ PCR cDNA synthesis it (Clontech Inc, USA) as per manufacturer’s protocol from total RNA itself. Subtraction was performed using PCR Select cDNA subtraction kit (Clontech,USA). Only forward library was prepared, as we are interested in up-regulated genes due to bruchid infestation. The subtracted cDNA clones were ligated into pGEM-T easy vector (Promega,USA) and transformed into JM109 competent cells. Transformed clones were selected on the basis of blue-white screening. The white colonies were selected from Luria-Bertini (LB) media supplemented with 100μg/ml ampicillin, 1mM% (W/V) IPTG and 80μg/ml (W/V) X-gal. A total of 277 recombinant cDNA clones were picked and plasmids from positive clones were recovered using a QIAprep Spin Miniprep kit (Qiagen). Plasmids were sequenced by M13 forward and reverse primers using Big Dye Terminator (Applied Biosystems, USA) to generate partial EST sequences on ABI 3730XL platform.

### EST sequence processing, assembly and annotation

EST sequences were scanned and trimmed to remove vector sequences using NCBI VecScreen Tool (http://www.ncbi.nlm.nih.gov/tools/vecscreen/). Low quality and short sequences were removed. High quality ESTs were assembled into contigs and singleton using the Contig Assembly Program, CAP3 following default parameters. Gene Ontology (GO) annotation of unigenes was performed using Blast2GOPro (http://www.blast2go.de). Sequences were annotated using BLASTX (https://blast.ncbi.nlm.nih.gov/Blast.cgi?PROGRAM) search in the GenBank NCBI database. Similarity with annotated sequence was considered to be significant having an E-value < 1× 10^−5^.

### Quantitative PCR (qPCR) of selected defense genes

Total RNA extraction from both oviposited and non-oviposited pods of mild tolerant black gram variety were performed with PureLink^™^ Plant RNA reagent (Ambion) and treated with DNAse-I (Sigma-Aldrich, USA) to eliminate traces of genomic DNA. The cDNA was synthesized using a PrimeScript^™^ RT Reagent Kit with gDNA Eraser (Perfect Real Time) (Clontech,USA) and real time PCR protocol was followed according to manufacturer’s instructions given in SYBR^®^ Premix ExTaq^™^ (Tli RNAse H Plus) (Clontech,USA). The thermal cycling conditions were carried out in Applied Biosystems StepOnePlus^™^ Real-Time PCR System (Applied Biosystems, USA) using the following program- 95°C for 30 sec followed by 40 cycles of 95°C for 5 sec and 60°C for 30 sec followed by a melt curve stage at 60°C for 1 min. Gene-specific primers were designed using Oligo Perfect Designer software program having a GC content of 55–60%, a T_m_>50°C, primer length ranging from 18–22 nucleotides and an expected amplicon size of 100-150bp. All quantitative real-time PCR experiments were performed twice using two biological replicates and each reaction was run in triplicate using the designed gene specific primers (S1 Table).

The relative gene expression levels were obtained by relative quantification (RQ) according to the 2^-ΔΔCt^ method [16].

## Results

### Generation of ROS in the developing seeds of black gram

**Fig 1:**
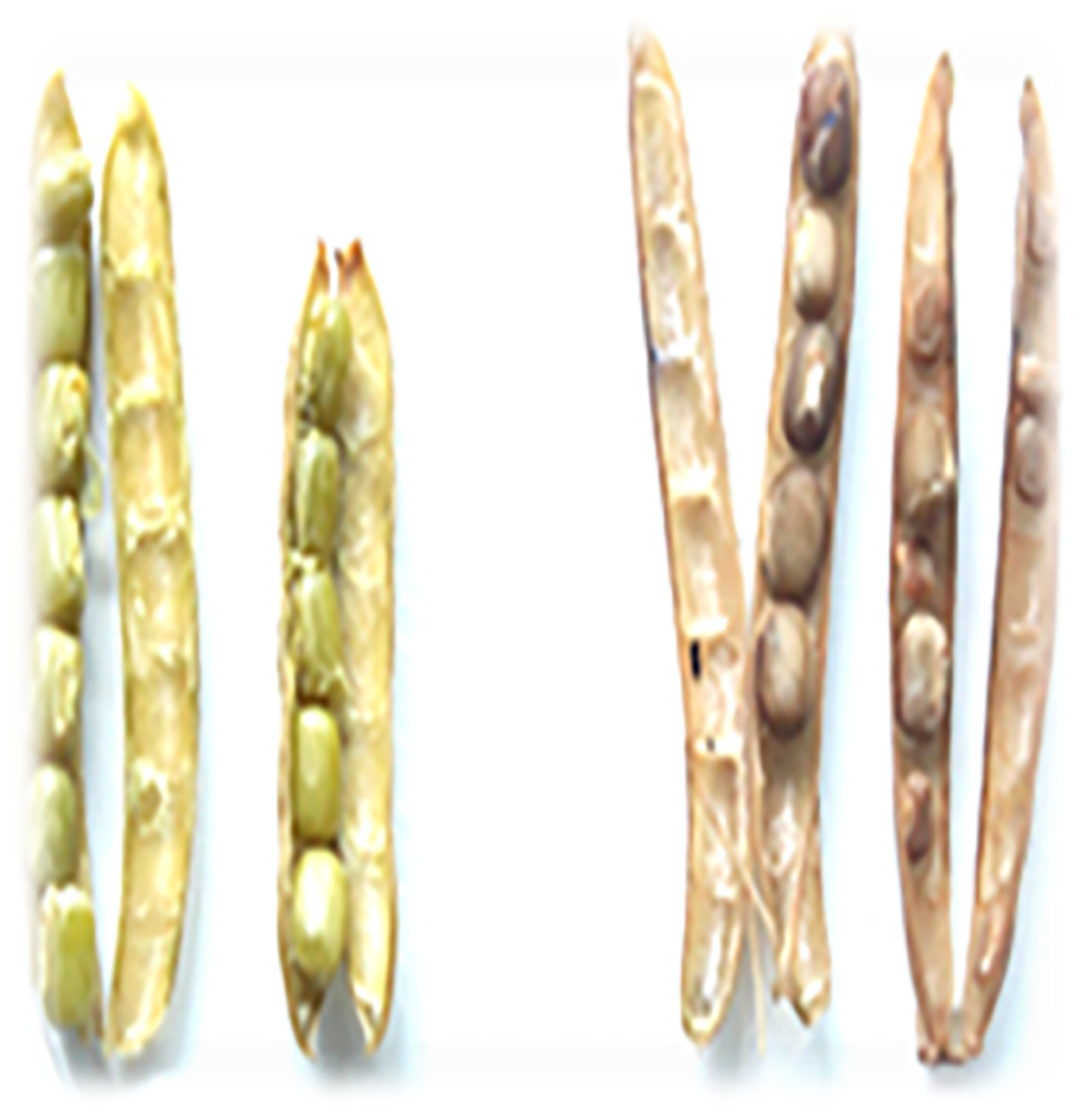
ROS accumulation in: A. Control pods of the mild tolerant plant. B. Oviposited pods of the mild tolerant plant after 7 days.

Reactive Oxygen Species (ROS) are associated with plant-insect interactions and there are several reports available on generation of ROS in response to stress. This acts as an indicator of defense response since there is crosstalk between ROS and hormone signaling during any stress [7]. The increment of the peroxidase activity which resulted in accumulation of H_2_O_2_ is evident in the response to bruchid beetle egg laying which lead to discolouration of the pod wall as well as the developing seeds inside in comparison to the green pod wall and seeds of non-oviposited control (Fig 1A and 1B).

### Generation of SSH Library and sequencing

To unravel genes involved in defense against bruchid, total RNA was isolated from the immature seeds of the pods oviposited by bruchids. Oviposited sample yielded a total of 1.3μg/μl and non-oviposited sample around 0.8μg/μl. Approximately 1 μg each from oviposited sample and non-oviposited sample was taken for construction of the subtractive cDNA library.

A total of 277 cDNA clones from the mild tolerant (IC 8219) cultivar of black gram were picked from the SSH library and sequenced after PCR amplifying with M13 primer. Sequences obtained were trimmed to remove vector sequences from ESTs using VecScreen and ESTclean tool of NCBI. A summary of unigene statistics has been illustrated in Table 1. Trimming resulted in 244 good quality sequences which were assembled using CAP3 [17] program into 134 non-redundant unigene sequences. Of these, 106 were singletons whereas the remaining was assembled into 28 contigs. The unigene length ranged from 102–1156 base pair (bp) with average unigene size of 476bp. All the unique ESTs have been submitted to in the EST database of NCBI GenBank (Accession ID: JZ917400—JZ917643).

**Table 1:** An overview of unigene statistics.

Description	Mild tolerant variety
Number of sequenced ESTs	277
Number of ESTs after quality control	244
Number of unigenes	134
Number of contigs	28
Number of singletons	106
Number of annotated unigenes	134
Number of non-annotated unigenes	59
Average unigene size	476

### Unigene annotation based on gene ontology

**Fig 2:**
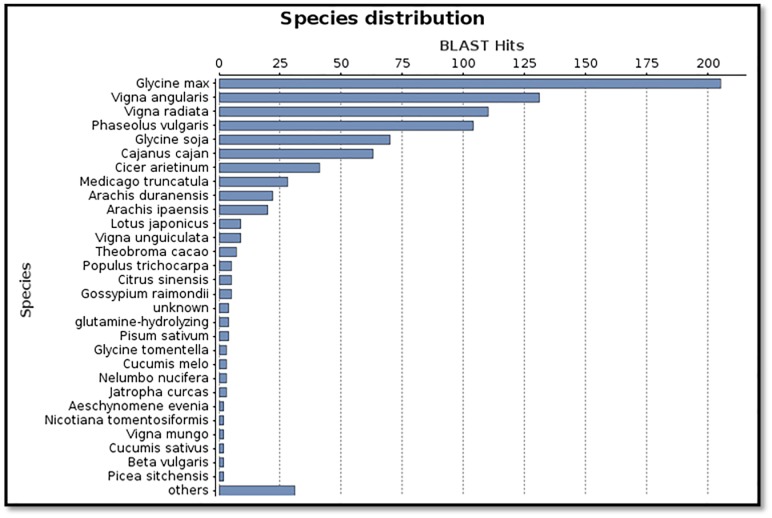
BLAST species level distribution of unigenes. Number of BLAST hits retrieved from NCBI databases. The majority of BLAST hits represents similarity with *Glycine max*, *Vigna angularis and Vigna radiata*.

Putative functions were assigned to unigenes to ascertain their role in different biological processes by gene ontology (GO) annotations using the Blast2GO program [18]. The software subjected all 134 unigenes to BLASTX against NCBI Nr (non-redundant) protein database. Homology search revealed that a majority of unigenes shared homology with *Glycine max* sequences followed by *Vigna angularis*, *Vigna radiata* and *Phaseolus vulgaris* at species level. In *Vigna radiata* too, stress related functional genomics studies have been very nominal and to date only Illumina sequencing has been used with this species for identification of EST-SSR markers [19]. The lack of BLAST hits with *Vigna mungo* against bruchid egg laying indicated the negligible amount of black gram stress related EST sequences in the database (Fig 2). So far, Mungbean Yellow Mosaic Virus (MYMV) infection related ESTs in *Vigna mungo* were identified [20].

### Functional annotation by gene ontology (GO)

**Fig 3:**
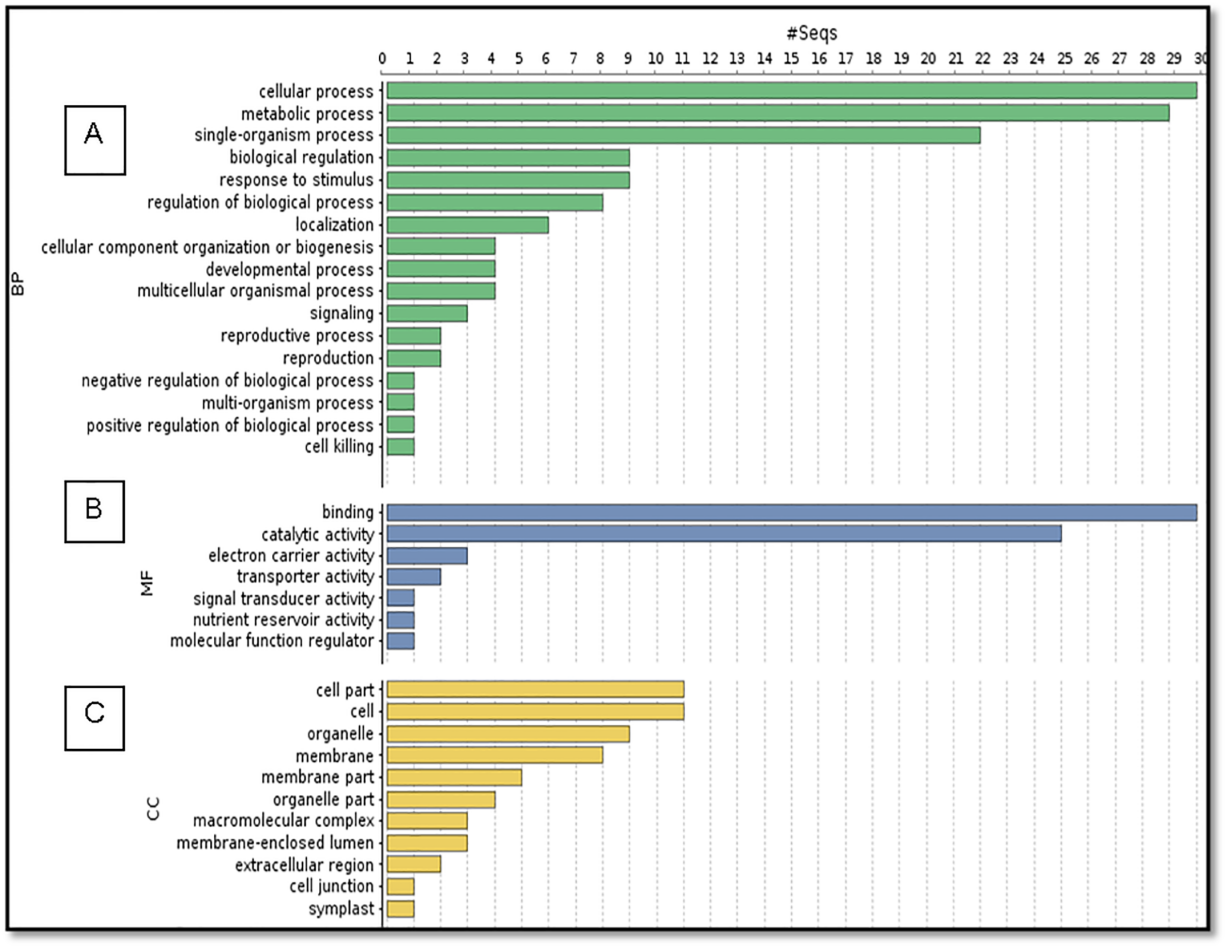
Functional categorization of GO terms. GO terms were distributed into (A) Biological function (B) Molecular Function (C) Cellular Component.

**Fig 4:**
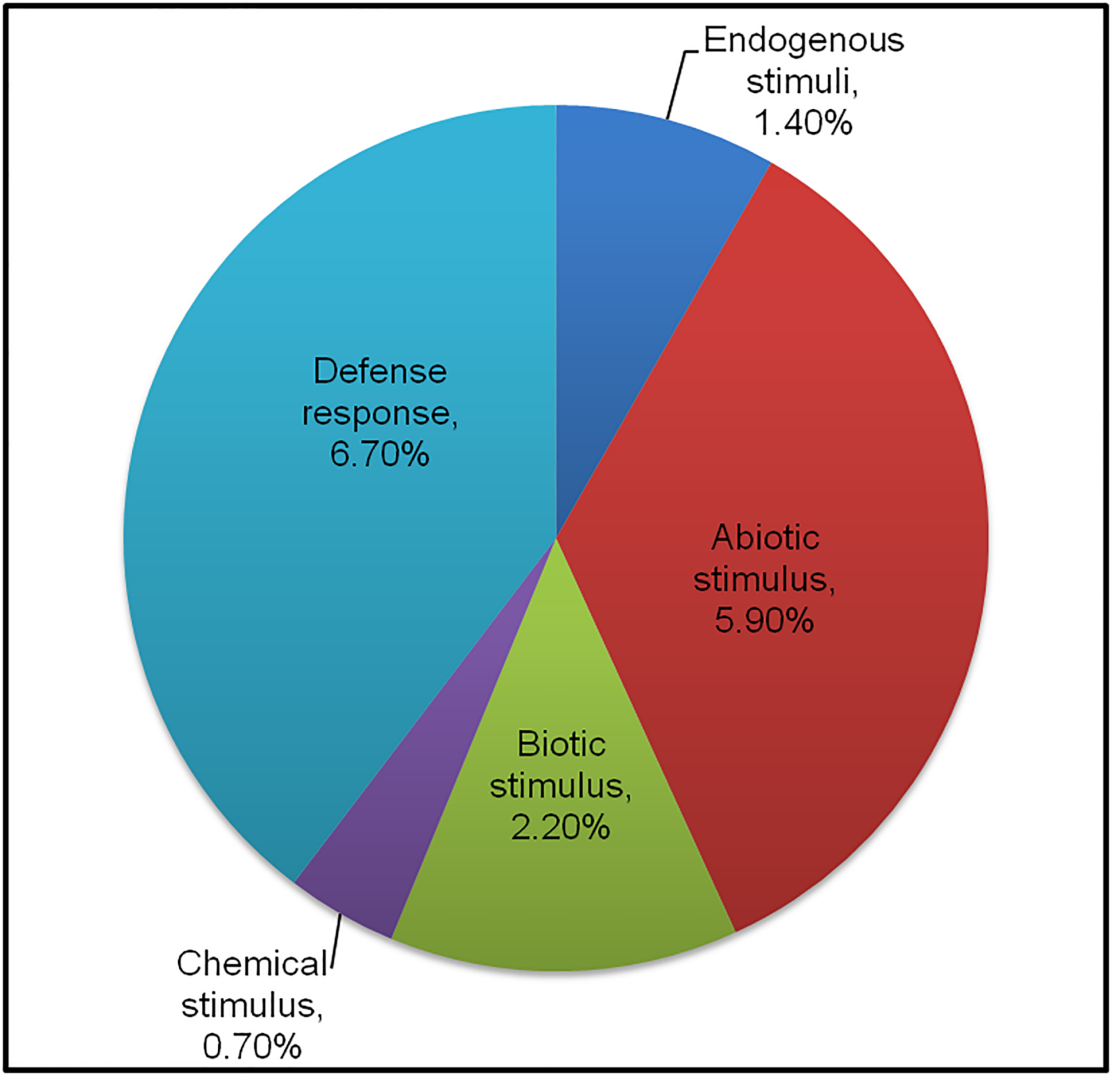
The pie chart represents sub-categories for “Response to stimulus” and their respective abundance in the SSH library under the biological process category.

Based on GO annotation, the unigenes were grouped into three categories viz. biological process, cellular component and molecular function (Fig 3). The unigenes with biological function participate in various processes and unigenes distributed in the metabolic process, cellular process, response to stimulus and biological regulation were found to be 22.3%, 23.1%, 12.6% and 5.9%, respectively. GO classification of differentially expressed unigenes revealed that 12.6% genes were involved in response to stimulus category, under which defense genes contributed around 6.7% and abiotic/biotic stimuli contributed 5.9%/2.2% respectively (Fig 4). In the forward subtractive library prepared, the most significant GO terms were associated with cellular process (GO: 0009987), metabolic process (GO:0008152) and response to stimulus category (GO:0050896). GO terms were also associated with signaling (GO:0023052) under biological process category.

### Functional categorization of unigenes

**Fig 5:**
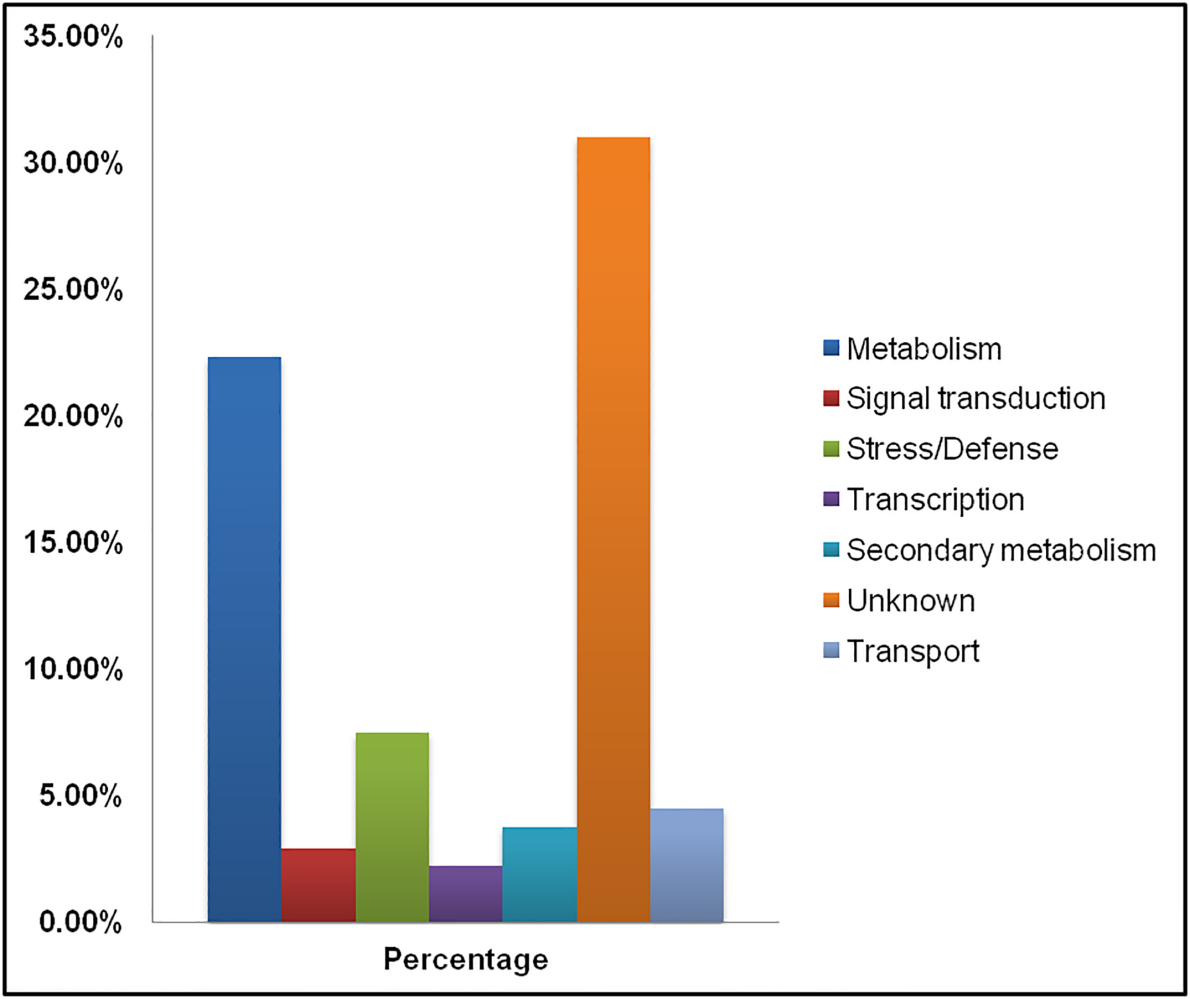
Differentially expressed ESTs after grouping into various functional categories. The horizontal bars represent percentage share of ESTs in the subtractive library.

Within each GO category, unigenes were further categorized into 7 different groups based on their putative functions (Fig 5). Unigenes were also annotated using KEGG (Kyoto Encyclopedia of Genes and Genomes KEGG) which showed enrichment of certain metabolic pathways, signaling pathway, biosynthesis of antibiotics. Presence of uncharacterized protein has a maximum abundance in our subtractive cDNA library leading to 31% of un-annotated sequences. In all, the majority of unigenes are involved in the metabolism (22.3%), namely amino acid metabolism pathways like asparagine synthetase (Acc. No.JZ917453), arogenate dehydratase (Acc.No.JZ917457), prephenate dehydratase (Acc.No.JZ917431), agmatine deiminase (Acc.No.JZ917504). Metabolism category was followed by stress and defense categories (7.46%) in which major defense genes like defensin (Acc. No. JZ917401), lipoxygenase (Acc.No. JZ917489), pathogenesis related protein (Acc. No. JZ917485) were identified. Other major functional groups include transport category (4.47%), secondary metabolite production (3.73%), signal transduction (2.29%) and transcription (2.23%) categories (Fig 5).

### Validation of defense related transcripts using quantitative PCR

We quantified relative expression of 20 unigenes from the SSH library of black gram in response to bruchid oviposition and compared with the non-oviposited seeds of the same genotype. Approximately 1.2μg of total RNA from oviposited and 1μg from non-oviposited sample was taken for qPCR analysis. The gene for elongation factor EF 1α was used as an internal control. Out of 20 genes, 12 up-regulated in the developing seeds from the pods oviposited by bruchid.

**Fig 6:**
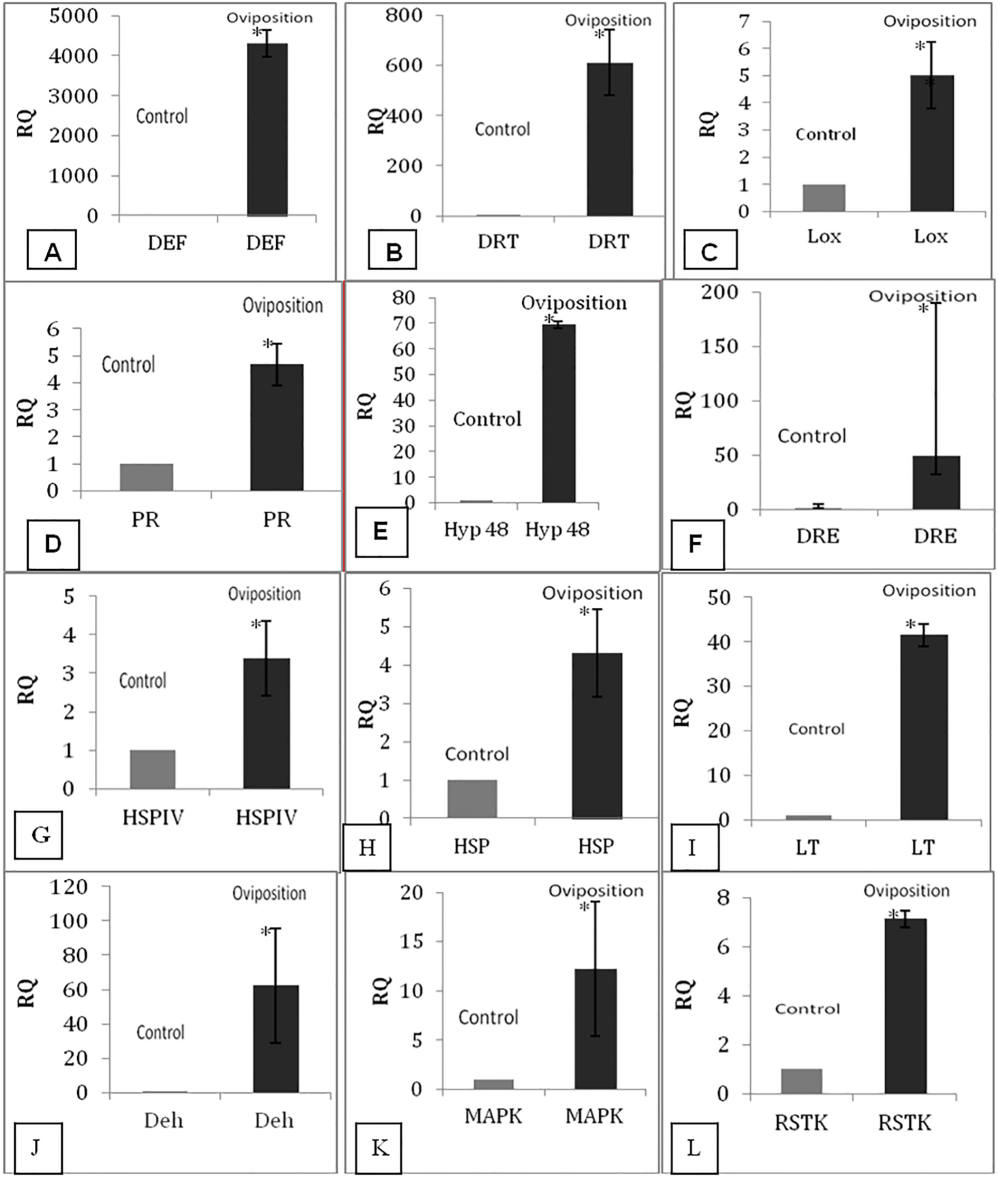
Expression levels of twelve defense genes up-regulated on insect attack. The expression levels were obtained by normalization with black gram EF1*α* gene. Expression analysis were done in two stages, control and oviposition after 7 days for (A) DEF (Defensin), (B) DRT (DNA damage repair toleration protein), (C) LOX (Lipoxygenase), D: PR (Pathogenesis related protein), (E) HYP48 (Hypothetical protein), (F) DRE (Dehydration responsive element transcription factor), (G) HSPIV (Heat shock protein), (H) HSP70 (Heat shock protein), (I) LT (low temperature induced protein), (J) DEH (Dehydrin), (K) MAPK (Mitogen activated protein kinase), (L) RSTK (serine threonine kinase like receptor). Bars represent mean ± standard deviation. * P value< 0.05, as determined by paired two-tailed student’s t- tests.

Interestingly, transcript coding for a defensin protein had maximum (>4000 fold) expression levels when compared with the un-infested controls (Fig 6A). The DNA damage repair toleration protein (DRT) showed 600 fold more expression than control (Fig 6B). We also validated major defense genes like lipoxygenase (LOX), pathogenesis related protein (PR) and found 5 fold and 4.6 fold increase in the levels of expression for LOX and PR, respectively (Fig 6C and 6D). A similar trend was also observed in the case of the hypothetical or un-characterized proteins with 70 fold increase in gene expression when compared with the control (Fig 6E).

A major drought responsive transcription factor, dehydration responsive element (DRE) had almost 50 fold higher expression than its corresponding control (Fig 6F). Other abiotic stress responsive genes like Heat shock protein IV, Heat shock protein 70, low temperature induced protein and dehydrin also showed 3.3, 4.3, 41 and 62 fold increase in gene expression, respectively (Fig 6G–6J). When the signal transduction genes were quantified, we found twelve fold and seven fold increase in mitogen activated kinases (MAPK) and receptor serine threonine kinase (RSTK) gene expression, respectively (Fig 6K and 6L).

## Discussion

The stage starting from insect egg laying to feeding by the larvae cause substantial damage to plants. Therefore, the plant’s defense mechanism not only gets activated by insect feeding, but also soon after egg laying or oviposition. The defense response to egg laying helps the plant to prepare its defense against feeding larvae even before larval hatching [21]. Such defense strategies may significantly contribute to a plant’s defensive arsenal against feeding herbivores [22–26]. The resistance mechanism of black gram against a viral pathogen (MYMV) showed up regulation of various defense related gene [20], however, the defense response to the bruchid egg laying was not elucidated. Therefore, we made an attempt to understand the underlying mechanism of bruchid resistance in a mild tolerant genotype of black gram.

We identified genes which are associated to defend against bruchids in black gram through an SSH library. The transcripts of the SSH library were differentially expressed and GO classification revealed that differentially expressed unigenes of black gram developing seeds were involved in defense response. This indicated that the resistance mechanisms in black gram developing seeds are governed by the transcriptional activation of genes that are involved in stress perception to actual response or adaptation [27, 28]. We know that the expression and the interaction of these genes are complex and diverse, and every gene involved forms part of a coordinated defense response network. Moreover, the speed and coordination of expression of these genes are vital for plant survival [29].

During the induced defense response, an increased accumulation of secondary metabolites, enzymes associated with cell-wall reinforcing, and proteins with toxic, anti-digestive, anti-nutritive activity are known to be associated with diverse plant-insect interactions [30,31]. Therefore, a forward suppression subtractive library of defense transcript gave us a clue on genes that are over expressed and their possible role in manifestation of defense response. The SSH library revealed induction of defense genes such as defensin, LOX, PR, DRE, HSP, including signal transduction genes such as receptor serine threonine kinase and mitogen activated kinases. During bruchid-black gram interaction, expression of enzymes associated with secondary metabolite pathways was also observed.

Appropriate perception and rapid response to stress conditions are important keys to elicit proper resistance to herbivore attack. In the resistant genotype, the regulatory mechanisms that confer tolerance, mostly involve the induction of stress-responsive genes [32]. Plants recognize herbivore associated molecular patterns (HAMPs) which often rely on receptors with amino-terminal extracellular domains implicated in elicitor recognition and protein-protein interactions; and a carboxyl-terminus intracellular kinase domain is involved in signal transduction [33]. The observation of a sevenfold increase in receptor serine threonine kinase (RSTK) expression in the developing seeds implicated that the receptor perceived the signals through elicitors, which might be bruchins in our case, in the developing seeds.

In most signal transduction cascades, regulatory molecules, such as protein kinases are also involved [34]. We found a twelve fold higher expression of Mitogen Activated Protein Kinases (MAPK) which suggests up-regulation of signal transduction pathway and its possible role in signal transduction during bruchid-black gram interaction. The activation of MAPK may be considered as an earliest signaling event after plant senses the insect egg attack. This is further substantiated by up-regulation of enzyme ERK1/2 (E.C.2.7.11.24) involved in Ras mediated m-TOR signaling pathway. Defense responses may include biosynthesis/signaling of plant stress/defense hormones, defense gene activation, Reactive Oxygen Species (ROS) generation and Hypersensitive Response (HR) cell death [35]. Thus, RSTK and MAPK mediated signal transduction may be involved in the downstream expression of various defense genes such as defensin, PR, LOX, HSP, etc.

The majority of plant defensins characterized, showed a constitutive pattern of expression, with an increase in expression in response to pathogen attack and wounding [36–39]. The gene, defensin, was found to express abundantly during black gram-bruchid interaction. The deduced nucleotide sequence of the defensin gene showed more than 90% identity to the nucleotide sequence of the well characterized defensins of *Vigna radiata*. A bacterially expressed mungbean (*Vigna radiata*) defensin have been earlier reported (VrCRP) to have exhibited both antifungal and insecticidal activities [40, 41]. Plant defensin from cowpea have been shown to be efficiently inhibiting α- amylases from bean weevils and Mexican bean weevil [42]. Therefore, defensin could be a potential candidate gene for resistance against bruchids.

Another plant defensive protein that is predominantly involved in plant defense against many stresses through octa-decanoid pathway is lipoxygenase (LOX), a group of anti-oxidative enzymes [43]. We found a fivefold increase in LOX expression in developing seeds during black gram-bruchid interaction. Similarly, pathogenesis related (PR) proteins are well established class of proteins that participates in defense mechanism. Such proteins accumulate in the cell and their concentrations are said to be high in and around the infected tissues. Production of PR proteins in the remote uninfected parts of plants can lead to the occurrence of Systemic Acquired Resistance (SAR), protecting the affected plants from further infection [44]. Up-regulation of PR-2 or β 1–3 glucanases in the present study demonstrated that they also played a role in black gram-bruchids interaction, because PR-2 are often expressed in response to wounding in other crops [45,46].

The role of dehydration responsive element transcription factor in abiotic stresses that includes salt, drought and extreme temperatures is well known. The connection between disease resistance and drought tolerance is well reported [47]. Transgenic *P*. *glaucum* plants overexpressing DREB TF suggested its cross talk with biotic stress related pathways [48]. Over-expression of the *OsDREB* led to an enhanced disease resistance against tobacco streak virus TSV in transgenic tobacco plants, apart from tolerance to various abiotic stresses [49]. Thus, upregulation of DREB TF by forty nine fold in the present study is interesting because this DREB might be one of the possible factors responsible for alleviating bruchid attack. We also found up-regulation of late embryogenesis abundant (LEA), dehydrin and heat shock protein, which are the most common defense gene against both biotic and abiotic stresses. Dehydrins (DHNs) constitute a distinct biochemical group of LEA proteins, which are known as group 2 LEA (or LEA II) proteins [50,51] or LEA-D11 proteins [52]. Similarly, heat shock proteins are also important for plant disease resistance as they have been reported to be important components of the hypersensitive response defense mechanism [53]. As such up-regulation of Hsp70 in our study is in concurrence with a tobacco HSP70 known to be involved in the unfolded protein response (UPR), which is caused by accumulation of unfolded or misfolded proteins in the lumen of the endoplasmic reticulum (ER) under various biotic and abiotic stress conditions [54].

Ubiquitination has also been demonstrated in plant signalling pathways, including those mediating responses to hormones, light, sucrose, developmental cues and pathogens [55]. The expression of E3 ubuiquitin ligase proteins in our study showed its involvement in the regulation of the signaling responses downstream of herbivore perception.

A number of enzymes were associated with specific biosynthetic pathways for secondary metabolites which participated to defend plants from stresses. Transcripts of enzymes involved in the synthesis of phenylpyruvate from prephenate, such as prephenate dehydratase (E.C.4.2.1.51) were found to be up-regulated in the developing black gram pods oviposited by bruchids. The prephenate dehydratase involved in the conversion of L-arogenate to phenylalanine during phenylpropanoid biosynthesis corroborates the fact that enzymes of phenylpropanoid pathway have been implicated in plant defense against pathogens and predators (viz. phenylalanine lyase) [56] and that it might be serving as a mediator of plant defense [57,58]. Furthermore, up-regulation of glutamine hydrolyzing synthase involved in alanine, aspartate and glutamate metabolism in black gram-bruchid interaction indicates that these pathways have been implicated in defense response as genes involved in glutamine metabolism have been earlier shown to be induced after exposure to virulent pathogen and pathogen derived elicitors [59]. The role of asparagine synthetase especially has already been implicated in defense response by tomato to *Botrytis cinerea* and has also been suggested that asparagine might be promoting *B*.*cinerea* pathogenesis [60]. Their activities likely to regulate downstream defense responses such as the activation of pathogenesis-related proteins (PR), the generation of reactive oxygen species (ROS), and the synthesis of the hormone salicylic acid (SA) that leads to the hypersensitive response (HR). Accumulation of aspartate-derived metabolites confers pathogen resistance by unknown mechanism(s) [57].

We have also found 41 unigenes with BLAST hits that could not be annotated with GO terms were categorized as unknown or un-characterized proteins as reported previously by other researchers [61]. A few hypothetical proteins (6 unigenes) were also identified in the library and qPCR expression of these proteins showed a very high level of expression in oviposited seeds. It appears to us that these hypothetical genes could be novel genes which may be involved in the immune response in black gram.

This study indicated that upon oviposition of eggs, black gram induced defense response by activating the entire signaling transduction cascade followed by expression of defense genes. However, further studies are required to understand both genetic and epigenetic effects of such signaling interactions.

## Conclusion

We looked at the molecular mechanism of black gram-bruchid interaction in the field and found that bruchid eggs on the pod wall induced alteration in transcript dynamics in the developing seeds of black gram. We found a total of 244 ESTs expressed differentially in black gram developing seeds after oviposition on the pod wall by bruchid beetle which were subsequently assembled into 134 unigenes. All genes were annotated by Blast2GOPRO and 12 genes were validated by qPCR. According to Blast2GO analysis, certain enzymes related to secondary metabolites, aromatic amino acid and primary amino acid metabolism were annotated. The study would be useful to isolate and clone defense gene (s) for further characterization of its eventual use in crop improvement.

## Supporting information

S1 Table List of primers used for qPCR analysis. Sequence information of gene specific primers used for qPCR analysis along with their Accession numbers allotted by GenBank.
(DOCX)
