# Supplementary material for: Bruchid egg induced transcript dynamics in developing seeds of black gram (Vigna mungo)
Source: PLoS One. 2017 Apr 27;12(4):e0176337. doi: 10.1371/journal.pone.0176337 (PMC5407641; doi:10.1371/journal.pone.0176337)
Supplement: S1 Table — Sequence information of gene specific primers used for qPCR analysis along with their Accession numbers allotted by GenBank. (DOCX) [file pone.0176337.s001.docx]

# Supporting Information

# S1 Table: List of primers used for qPCR analysis. Sequence information of gene specific primers used for qPCR analysis along with their Accession numbers allotted by GenBank.

| **Primer name** | **Primer Sequence** |
| --- | --- |
| Defensin (Acc. No. JZ917401) | F: ACACCGGAAATCATCTCTGC |
|  | R: GGCAAAGACTTGCGAGAATC |
| Dehydrin 19 (Acc. No. JZ917444) | F: GAACCAAGTGGGCTGAACAT |
|  | R: ATGGAGCCTTGCAGTCACTT |
| PR 2 (Acc. No. JZ917485) | F: ATCAGGGATTGGCCAAAAGG |
|  | R: TCCTACCACGGCAAAGGAGA |
| Lox (Acc. No. JZ917489) | F: TCCTCTGCAGGCAGAAAAAC |
|  | R: AACAAGGCTTATGCCACCAG |
| HSP iv (Acc. No. JZ917618) | F: AGGACCCCATTGTCGAGCTT |
|  | R: AAGGGGATCACTGGCACAGA |
| Heat shock protein 70 (Acc. No. JZ917428) | F: TGCAGATTCCTTCCAACTCC |
|  | R: TTGAGAAGGCTGTGGAGGAT |
| MAPK (Acc. No. JZ917425) | F: GGGCCATGTAAGGGTGTTGA |
|  | R: TCTCCTGGAACCCCCTTTTC |
| Receptor serine threonine kinase (Acc.No.JZ917609) | F: GAGTCAATGGGCCAGTCTGC |
|  | R: ATGCAACAAGGGGCACCA |
| DNA damage repair toleration protein 100  (Acc. No. JZ917465) | F: AATCTGAAAGGGGCCATACC |
|  | R: GATGATCGAAGGGAGATCC |
| Dehydration responsive element transcription factor (Acc. No. 917435) | F: GTTTGGCGGTGGCACTGTT |
|  | R: CAACTTCCTACCGTGCCCAGA |
| Low temperature 65 protein  (Acc. No. JZ917464) | F: CCTCTGATTCGGTTACCTTCC |
|  | R: TGCATAAGAAGGAGCCAGTG |
